# Anemia and nutritional aspects in adolescent athletes: a cross-sectional study in a reference sport organization

**DOI:** 10.1590/1984-0462/2022/40/2020350

**Published:** 2021-10-04

**Authors:** Flávio Diniz Capanema, Joel Alves Lamounier, José Geraldo Leite Ribeiro, Cláudio Olívio Vilela Lima, Alan Rodrigues de Almeida Paiva, Patrícia Ribeiro Quadros, Nádia Sachie Koyama Ferreira, Tatiane Soares de Almeida, Nicolly Carla Machado Santos

**Affiliations:** a Faculdade da Saúde e Ecologia Humana, Vespasiano, MG, Brazil.; b Universidade Federal de São Jõao del-Rei, São João del-Rei, MG, Brazil.; c Sports Science Center of Minas Tênis Clube, Belo Horizonte, MG, Brazil.

**Keywords:** Anemia, Hemoglobin, Prevalence, Adolescents, Athletes, Anemia, Hemoglobina, Prevalência, Adolescentes, Atletas

## Abstract

**Objective:**

To assess the association between anemia and nutritional aspects in adolescent athletes from a large sport club.

**Methods:**

This is a cross-sectional study, involving 298 athletes aged between 10 and 17 years, submitted to measurement of skin folds, weight and height, and collection of capillary blood in duplicate to determine hemoglobin values. It was carried out in a random sample composed of athletes from eight sport modalities.

**Results:**

Regarding nutritional status, 10.1% of athletes were overweight based on body mass index and 70 (23.5%) athletes had a percentage of body fat classified as high or very high. The prevalence of anemia was 16.4%, being more prevalent in judo (37.1%), basketball (34%) and futsal (20.5%) athletes. Low hemoglobin levels were significantly associated with shorter stature (p=0.006).

**Conclusions:**

There was a significant association between anemia and short stature, suggesting that the athlete's height-weight development may be affected in suboptimal conditions of oxygen distribution.

## RESUMO

**Objetivo:**

Avaliar a associação entre anemia e aspectos nutricionais em atletas adolescentes de um clube desportivo de grande porte.

**Métodos:**

Trata-se de estudo de corte transversal que envolveu 298 atletas com idades entre 10 e 17 anos, submetidos à aferição de dobras cutâneas, peso e altura, além de coleta de sangue capilar em duplicata para a determinação dos valores de hemoglobina. Foi realizado em amostra aleatória composta de atletas oriundos de oito modalidades esportivas.

**Resultados:**

Em relação ao estado nutricional, 10,1% dos atletas apresentaram excesso de peso com base no índice de massa corpórea e 70 (23,5%) atletas estavam com porcentagem de gordura corporal classificada como alta ou muito alta. A prevalência de anemia foi de 16,4%, mais prevalente nos atletas do judô (37,1%), basquete (34%) e futsal (20,5%). Níveis baixos de hemoglobina foram associados à menor estatura (p=0,006).

**Conclusões:**

Observou-se associação significativa entre anemia e baixa estatura, o que sugere que o desenvolvimento pôndero-estatural do atleta pode ser afetado em condições subótimas de distribuição de oxigênio.

## INTRODUCTION

Adolescence, as defined by the World Health Organization (WHO), is the period of life between 10 and 19 years old,^1^ marked by intense organic, psychological and social transformations — — the moment of the body crossing the line between childhood and adulthood. At this point, the singular characteristic of increase in speed of body growth — the pubertal spurt — requires a significant increase in the demand for nutrients to support it.^2^ Anemia due to iron deficiency and obesity are the most common nutritional disorders worldwide, and the period of adolescence is of great vulnerability to such diseases.^3^

The WHO defines anemia as a condition in which the hemoglobin (Hb) content in the blood is below normal for age, as a consequence of deficiency of one or more essential nutrients.^4^ Deficiency anemia causes a marked decrease in myoglobin levels, with a consequent reduction in the aerobic capacity of the muscle fiber.^5^ This process may result in less strength of the muscle groups in the mechanical traction in the bones. In the case of individuals who are in the process of bone maturation, this strength plays a fundamental role in remodeling and increasing bone mass. Thus, it is not uncommon for athletes to be referred to the health service complaining of training difficulties, asthenia and decreased sports performance.^5^ ^,^ ^6^

Obesity has an increasing prevalence among adolescents in recent decades, and the consequences can occur in the short and long term.^7^ ^,^ ^8^ The literature has shown that obesity at this stage is associated with the onset of diseases such as arterial hypertension, diabetes, dyslipidemia and early atherosclerotic events in adult life.^3^ ^,^ ^7^ ^,^ ^8^ In addition, the low-grade inflammatory stage of excess body fat can lead to changes in iron metabolism, with tissue overload, myoglobin degradation, decreased mobility and decreased serum iron for hematopoiesis.^9^

Considering that the most common nutritional problems in adolescence — overweight and anemia — can compromise athletes in this age group, this study was carried out to evaluate the association between anemia and nutritional aspects in adolescent athletes of a large club with different sport modalities.

## METHOD

This is a descriptive comparative cross-sectional study carried out with under-20 athletes from Minas Tennis Club. The sample was obtained from a total of 912 individuals, distributed in eight sport modalities of the basic categories of Minas Tennis Club, a sports and social association based in Belo Horizonte (MG), which has about 73 thousand members and 471 thousand m^2^ of area, divided into four Units. The club traditionally trains high-performance athletes participating in national and international competitions and, in 2019, it had about one thousand federated athletes, 900 of them in training.

All participating athletes are part of the teams in base category, distributed in different sports and ages. The athletes have no employment contract with the club in this age group, so they are classified as amateurs. Each sport modality has a specific training periodicity and weekly variations depending on the game scale, the team's performance, the opponent and the training of new skills. However, all modalities in this age group have a minimum of three workouts per week and a maximum of five, with an average of two hours a day.

Athletes’ dietary and nutritional assessments are made annually through dietary questionnaires and anthropometric measurements by qualified professionals. Based on this screening, those with unsatisfactory questionnaires or nutritional disorders are individually monitored by the club's professional nutritionist.

Most of the club's athletes have medium to high socioeconomic status, given the need for fees for training and access to the club. However, a small portion of athletes with low socioeconomic status hold a scholarship for sports performance.

The sample calculation was based on the sample universe, which reflects the prevalence of iron deficiency anemia among adolescents, around 20%.^4^ Assuming the sample error of 5% and the 95% confidence interval (95%CI), the following formula 1 was used:

(1) $$n=\frac{\text{p}(1-\text{p})1,96^{2}}{{\text{E}}^{2}}=246$$

To that number, 20% was added for possible losses. Thus, the final number of 295 was proposed.

For the sample selection, athletes over the age of 10 years or less than 17 years, 11 months and 29 days who presented the terms of free and informed consent signed by their parents or guardians for authorization to participate in the research were included. Athletes known to have chronic or acute diseases on the day of the examination were excluded. Those with no difference between the hemoglobin values obtained in duplicate (Hb1–Hb2) ≥1.5g/dL did not enter the study, which allows greater reliability of the sample and less consumption of disposable material. In addition, adolescents with insufficient cognitive discernment to spontaneously understand and accept interventions or refuse to participate in the research were excluded from the study.

The athletes selected for the study were referred to the campus by the coaches responsible for each sport, in order of arrival, before the start of training. Thus, there was a random collection, since the sports campus has simultaneous training in different modalities, due to the large number of gymnasiums present, and the collections were made on different days during the club's operation.

The variable age was determined by the birth date of each athlete, and the anthropometric variables were provided in a standardized way by Minas Tennis Club. The body mass index (BMI) was obtained by calculating the weight (kg) divided by the square of the height (m) of the athletes. Anthropometric measurements were obtained with the participation of previously trained evaluators from the club itself. The electronic anthropometric scale with a coupled ruler graduated up to 2 m (Welmy®/Brazil) was used. In addition, measurements of the tricipital and subscapular skinfolds were performed with a scientific analogue plicometer (Cescorf®/Brazil). Data was collected from 224 athletes, as 74 did not show up at the skinfold collection session.

Blood collection was carried out on the club premises by the team of researchers. The mean hemoglobin values were determined based on the collection of two drops of blood — duplicate sample — by puncture of the left index finger, using sterile and individualized disposable material. The drops obtained, coded as Hb1 and Hb2, were collected directly by means of a microcuvette that contained reagent material in dry chemistry and then forwarded for reading in a Hemocue beta-hemoglobinometer, model HB201, properly calibrated and in accordance with the manufacturer's instructions. The sensitivity of the equipment ranges from 75 to 91% and the specificity from 88 to 100%.^10^

The participants’ personal data were coded and recorded in a specific form, keeping confidentiality, as well as information regarding the variables age, sex, sport modality, weight, height, skinfolds, BMI, Z score BMI/age, Hb1 and Hb2. The data obtained were stored in an Excel® spreadsheet and analyzed using the IBM Statistical Package for the Social Sciences (SPSS) software, version 25.0.

Anemia was defined as hemoglobin level below the reference values adjusted for age and sex. The cutoff points used for the definition of anemia were based on the WHO recommendation. Therefore, individuals aged 5 to 11 years with hemoglobin less than 11.5g/dL were considered anemic; as well as aged 12 to 14 years below 12g/dL; over 14 years below 12g/dL for women and below 13g/dL for men.^4^

When assessing nutritional status, the Ministry of Health's recommendation on the use of anthropometric indicators for children and adolescents was used, so we calculated the percentile and the BMI Z score, using the standardized coefficients with information about sex, age, weight and height reported.^1^ ^,^ ^4^ The nutritional status classifications were based on the Food and Nutrition Surveillance System (SISVAN) for each anthropometric index, in line with the WHO.^2^

Bearing in mind the constant changes in weight and height throughout the adolescents’ development, Lohman's equation^11^ was used to calculate body fat because it is specific for adolescents aged 7 to 16 years. The equation 2 is defined as:

(2) $$\%\text{BF}=1,35\;(\text{TF}+\text{SF})-0,012\;{\left(\text{TF}+\text{SF}\right)}^{2}-\text{C}$$

Where:

% BF (body fat);

TF (tricipital fold);

SF (subscapular fold);

C (adjustment constant for sex and age).

The chi-square test was used to test the association between a categorical variable and two or more independent groups. In the evaluation of normality distribution for continuous variables, the Kolmogorov-Smirnov and Shapiro-Wilk tests were applied. The Student's t test was used to test the relationship between a continuous parametric variable and two or more independent groups, and, for variables with non-parametric distribution, the Kruskal-Wallis test was adopted.

Then, we performed a multivariate analysis using the logistic regression model, establishing anemia as a dependent variable. Correlation analyses were performed using the Cox-Snell and Nagelkerke summary model.

The parameters adopted in the study were 95%CI and significance level of 5%, and a relationship with a p-value less than or equal to 0.05 was considered statistically significant.

As this research involves human beings, it was developed in compliance with the ethical criteria of Resolution 466/12 of the National Health Council. In addition, the study was submitted to and approved by the Research Ethics Committee of the Faculty of Health and Human Ecology of Vespasiano, Minas Gerais, under opinion number 3,106,087, and Certificate of Presentation for Ethical Appreciation (CAAE) number 02891218.0.0000.5101, dating of November 20, 2018.

## RESULTS

Initially, 332 adolescent athletes were selected at random to compose the study. Of these, 19 were excluded because they did not have authorization from their parents or guardians at the time of the examination and nine others due to differences in hemoglobin (Hb1–Hb2) ≥1.5g/dL. There were six losses due to problems with filling in the respective forms with insufficient data. Thus, the final sample consisted of a total of 298 subjects. Of these, 213 (71.5%) were males and 85 (28.5%) females, with an average age of 172.3±20.3 months and a median of 172.9 months.

The classification of the nutritional status of adolescent athletes was determined by the index of the BMI Z score/age^4^ (Table 1). A prevalence of 10% (n=30) of overweight was found, with only one case classified as obesity.

**Table 1:** Nutritional status of athletes from a sports club in Belo Horizonte (MG) according to the Z score and body mass index/age

Nutritional status		Frequency	%
Z Score			
	<−3	01	0.3
	≥−3 and <−2	03	1.0
	≥−2 and <−1	12	4.0
	≥−1 and ≤+2	252	84.6
	>+1 and ≤+2	29	9.7
	>+2 and ≤+3	01	0.3
	Total	298	100
BMI for age			
	Thinness	04	1.3
	Eutrophy	264	88.6
	Overweight	29	9.7
	Obesity	01	0.3
	Total	298	100

BMI: body mass index.

Regarding height/age index^4^, 98.7% (n=294) of the individuals were normal, and only 1.3% (=4) met the criteria for short stature.

As for the distribution of athletes by sport, most of them practiced futsal and swimming, 22.8% ech (n=68).

**Graph 1:**
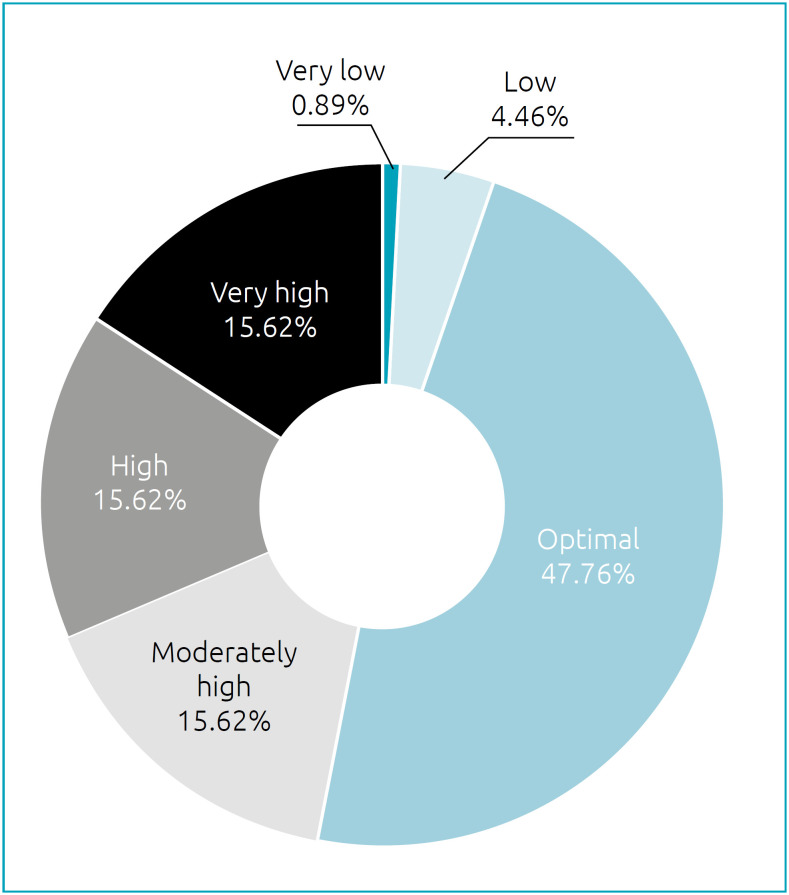
Distribution of athletes per body fat.

The adiposity index obtained was stratified by Lohman's classification^11^ for sex and age of each athlete. The frequency is illustrated in Graph 1.

For the variable gender, there was a statistically significant difference (p=0.000) between the groups comparing body fat percentage (%BF). Men presented, in their majority, an excellent fat percentage (52.6%), while women presented a very high %BF (32.7%).

Regarding the continuous variables, weight, height and BMI showed a significant difference for %BF, which is shown in Table 2. In the analysis of weight and BMI, individuals with higher values presented percentages considered high and very high.

**Table 2:** Analysis of athletes’ body fat classification according to body mass index, height and sex (Belo Horizonte, MG).

Continuous variable	Classification %BF	n	Mean station	Mean	p-value*
BMI	Very low	2	38.0	17.3	<0.001
	Low	10	32.4	16.8	
	Optimal	107	92.5	19.2	
	Moderately high	35	121.2	20.3	
	High	35	141.5	21.4	
	Very high	35	163.2	22.4	
Height	Very low	2	14.0	1.4	0.007
	Low	10	47.1	1.5	
	Optimal	107	115.0	1.7	
	Moderately high	35	113.7	1.7	
	High	35	118.3	1.7	
	Very high	35	122.1	1.7	

Categorical variable	Classification %BF	n Males	n Females	Total	p-value
Sex	Very low	0	2	2	<0.001
	Low	9	1	10	
	Optimal	92	15	107	
	Moderately high	29	6	35	
	High	26	9	35	
	Very high	19	16	35	

BMI: body mass index; BF: body fat

* Kruskal-Wallis test.

The prevalence ratio between individuals with high %BF and anemia was not statistically significant (p=0.22).

The analyses of mean values of digital hemoglobin showed average hemoglobin of 13.8g/dL for Hb1, Hb2 and mean Hb in athletes. The mean Hb ranged from 10.4g/dL to 17.4g/dL. The total prevalence of anemia found in adolescent athletes was 16.4% (n=49).

Still in relation to mean hemoglobin, the variables gender (p=0.494), age (p=0.859) and BMI/age (p=0.341) did not present a significant association with anemia. The variable sport modality showed a significant relationship (p<0.001) with the Hb values, indicating a higher prevalence of anemia in athletes practicing judo (37.1%), basketball (34.5%) and futsal (20.6%). These data are shown in Table 3.

**Table 3:** Mean hemoglobin in adolescent athletes according to sex, modality of sport and body mass index.

	Category	Hemoglobin classification			p-value
		Low	%	Total	
Sex	Male	37	17.4	213	0.494
	Female	12	14.1	85	
	Total	49	16.4	298	
Modality	Basketball	10	34.5	29	<0.001
	Futsal	14	20.6	68	
	Artistic gymnastics	02	16.7	12	
	Trampoline gymnastics	01	12.5	08	
	Judo	13	37.1	35	
	Swimming	03	4.4	68	
	Sneakers	0	0.0	13	
	Women's Volleyball	05	13.5	37	
	Men's Volleyball	01	3.6	28	
	Total	49	16.4	298	
BMI for age	Thinness	01	25.0	04	0.341
	Eutrophy	40	15.2	264	
	Overweight	08	27.6	29	
	Obesity	0	0.0	01	
	Total	49	16.4	298	

BMI: body mass index.

The variables weight (p=0.014) and height (p=0.001) had a significant relationship with Hb. Anemic individuals were associated with lower weight and height. The Student's t test was 0.014 in the association between low weight and anemia. In addition, it resulted 0.001 in the association between short stature and anemia.

In the regression model, the variables height (p = 0.001) and sport modality (p<0.001) were statistically significant, as shown in Table 4.

**Table 4:** Final logistic regression model based on the presence of anemia.

Variables in the equation associated with the presence of anemia						
	B	SE	Wald*	Degrees of freedom	p-value	ExpB
Height	3.250	1.176	7.633	1	0.006	25.794
Sport	0.208	0.070	8.805	1	0.003	1.239

B: constant; SE: standard error; ExpB: *exponent of the constant B*

* Wald test.

Regarding the variables that are part of the model, both had a positive B coefficient, showing a relationship in which taller athletes tend to have normal hemoglobin. The B coefficient of sport modalities, on the other hand, indicated a small effect on the dependent variable anemia.

Thus, the most adequate model was obtained to explain the occurrence of anemia. According to the Cox-Snell summary model, 6.8% of the variations can be explained by the set of independent variables. The Nagelkerke model was able to explain 11.4% of the variations recorded in the dependent variable — mean Hb —, without a relevant degree of adjustment.

## DISCUSSION

The data in this study point to the absence of an association between anemia and overweight in adolescent athletes. The prevalence of anemia was lower than expected for the adolescent population in general, but there was an association between anemia and short stature.

Anemia is recognized as the biggest nutritional problem among adolescents in general. It is caused by micronutrient deficiency, particularly iron deficiency and, depending on the context, it is also associated with malnutrition, obesity and other comorbidities.^12^ In addition, the occurrence of anemia in this phase is related to intrinsic factors of adolescence, such as the increased demand caused by the pubertal spurt, inadequate eating habits and menstrual irregularities.^13^ A Brazilian study pointed out a prevalence of 31% of anemia among adolescents, regardless of gender.^13^ This data was close to that found in another study regional, in which 272 adolescents aged between 10 and 18 years were evaluated and a prevalence of anemia of 31.2% was reported, with no significant difference between genders.^12^

In our study, the prevalence of anemia in adolescent athletes was 16.4%. In a survey conducted in the north of the country, in a lower economic class location, the prevalence was 41.7%.^14^ Other studies highlight the lower prevalence of anemia in athletes when compared to the general population. This may be related to healthier lifestyle habits, with a balanced diet and a more routine monitoring of health status in these individuals.^15^ ^,^ ^16^ This was also observed in a Japanese study whose objective was to assess the prevalence of anemia in the total population of athletes of a university compared to non-athletes, with a lower prevalence in athletes: 8.5% and 19.8%, respectively.^16^

About 50% of an individual's weight and 20–25% of their height are acquired in adolescence, and nutrition at the population level is a highly significant determinant factor of the variability of this process.^17^ This is because the secretion of gonadal hormones can be inhibited by insufficient amounts of nutrients, delaying the onset of puberty development and, therefore, compromising height gain.^18^ ^,^ ^19^

In addition to the hormonal importance in adolescent growth, nutritional deficiencies contribute to the decrease in muscle aerobic capacity due to a low concentration of myoglobin.^5^ The weakened muscle decreases the mechanical strength on the bones, which is essential for the increase in bone mass resistance and its remodeling.^6^ ^,^ ^7^ An imbalance at this moment of development can lead to an increase in fractures due to bone fragility and difficulty in growth, among other pathologies.^5^ ^-^ ^7^

In this study, we found a direct relationship between lower hemoglobin levels and shorter stature, and it can be inferred that anemia can be an interference factor in the optimum height growth in these adolescents.

The study by Opoku-Okrah et al.^20^ assessed anemia among futsal players and reported a reduction in hematological parameters, including hemoglobin concentration, hematocrit and red blood cell count, pointing out that such changes can lead to a condition known as “sport anemia” or “pseudoanemia”.^20^ In the present study, there were a large number of athletes practicing futsal and adolescents in this modality were significantly related to the higher frequency of low hemoglobin. Thus, further studies are needed to understand and confirm or not this association.

The most prevalent type of anemia in adolescents is of deficiency anemia, especially iron deficiency, which is responsible for fatigue and decreased physical performance.^16^ ^,^ ^21^ The justification would be related to the loss of iron through sweat, gastrointestinal bleeding and hemolysis caused by stress, in addition to repeated injuries in the lower limbs of athletes.^16^ ^,^ ^22^ ^,^ ^23^ The consequences of this deficiency can take place in the short and long term, affecting appetite, immune response, learning capacity and neuropsychomotor development.^24^ ^-^ ^26^ Thus, in athletes, anemia assumes clinical importance for both health and performance.^22^ ^,^ ^24^ ^-^ ^26^

According to Giudice et al.,^27^ in overweight/obesity, a low-grade inflammation occurs in different tissues, and some studies have recently supported the idea that iron deficiency could be one of the comorbidities associated with the typical low-grade inflammation in obese patients. However, the results of our study did not confirm the association between overweight/obesity and anemia in adolescent athletes.

It should be noted that this investigation has some limitations, including the predominance of males and certain sports in the sample, resulting from the non-interference of the research team on the referral of athletes at the time of selection.

In view of these findings, short stature in athletes may result from deficient levels of hemoglobin circulating in the period of development, and nutritional disorders are the main causes of this deficit in adolescents. In addition, frequent bone fragility disorders present in the adult athlete can be acquired in the period of weight-height growth. Thus, further studies on this stage of development are needed to ratify or not these associations and help to establish preventive measures.
